# Cardiac Safety in Breast Cancer Patients Receiving Pegylated Liposome Doxorubicin Sequential Anti-HER2 Monoclonal Antibody Therapy

**DOI:** 10.3389/fphar.2022.883600

**Published:** 2022-08-04

**Authors:** Ping Huang, Jia-huan Huang, Ya-bing Zheng, Wen-ming Cao, Xi-ying Shao, Jun-qing Chen, Yuan Huang, Guang-liang Li, K Sharma, Huan-huan Zhou, Xiao-jia Wang, Hong-chuan Jin, Zhan-hong Chen

**Affiliations:** ^1^ Department of Breast Medical Oncology, The Cancer Hospital of the University of Chinese Academy of Sciences (Zhejiang Cancer Hospital), Institute of Basic Medicine and Cancer (IBMC), Chinese Academy of Sciences, Hangzhou, China; ^2^ Department of Internal Medicine, Second Clinical Medical College of Zhejiang Chinese Medical University, Hangzhou, China; ^3^ ICardioOncology (Official Cardio-Oncology Organization in China), Shanghai, China; ^4^ Sir Run Run Shaw Hospital, Zhejiang University School of Medicine, Hangzhou, China

**Keywords:** cardiotoxicity, early breast cancer, HER2-positive, pegylated liposomal doxorubicin, trastuzumab

## Abstract

**Background:** Cardiotoxicity associated with the sequential use of anthracyclines followed by trastuzumab is common in adjuvant therapy of patients with HER2-positive early breast cancer (eBC). However, the cardiac safety of trastuzumab concurrent with pegylated liposomal doxorubicin (PLD) is relatively less studied.

**Method:** Clinical data of patients with HER2-positive eBC treated with PLD and cyclophosphamide (PLD-C) followed by taxanes plus trastuzumab ± pertuzumab (TH or TPH) who then completed standard anti-HER2 treatment for 12 months from June 2012 to August 2021 were retrospectively collected. The primary endpoints were clinical and subclinical cardiotoxicity.

**Result:** In total, 70 eligible patients were enrolled. Among them, 55 patients (78.6%) received PLD-C → TH and 15 patients (21.4%) received PLD-C → TPH. The median follow-up time was 41.8 months. Until August 2021, only two patients had recurrent or metastatic diseases, with 2-year and 5-year disease-free survivals of 98.6% and 96.8%, respectively. Clinical cardiotoxicity occurred in six patients (8.6%), and all of them had an absolute decline of ≥16% from baseline left ventricular ejection fraction (LVEF) but not below the lower limit of normal (LLN = 50%). Subclinical cardiotoxicity events occurred in 17 patients (24.3%), and all of them had absolute declines of ≥10% and <16% from baseline LVEF but not below the LLN. No patients were interrupted from treatment, and all patients completed anti-HER2 treatment for 12 months. The sharpest decrease in LVEF was observed at 18 months after the start of PLD treatment. The cumulative incidences of clinical and subclinical cardiotoxicity were 9.8% and 28.3%, respectively. In the univariate analysis, body mass index, age, left chest wall radiotherapy, and ongoing cardiovascular risk factors were not significantly associated with clinical or subclinical cardiotoxicity (*p* > 0.05). No patients had congestive heart failure or death caused by PLD or anti-HER2 treatment.

**Conclusion:** The sequential use of PLD and trastuzumab showed a lower incidence of clinical cardiotoxicity, presented as asymptomatic decreased LVEF, compared with the results obtained in previous clinical studies using conventional anthracycline, taxanes and trastuzumab. The study regimen demonstrated good cardiac tolerance and is an alternative strategy for cardioprotection in patients with HER2-positive eBC.

## Introduction

Breast cancer is the most common type of malignant tumor and the leading cause of death in women worldwide (Sung et al., 2021). The use of chemotherapy has significantly improved both mortality and morbidity outcomes in breast cancer patients. Anthracyclines are widely used in the treatment of hematological malignancies and solid tumors, with powerful antitumor effects and indispensability (Nicolazzi et al., 2018). However, cardiotoxicity is a serious side effect of these kinds of drugs, which limits their clinical application (Schwentner et al., 2016). Pegylated liposomal doxorubicin (PLD) is a new dosage form of doxorubicin confined in liposomes, which can form a stable three-dimensional structure when polyethylene glycol is grafted onto the surface (stealth liposome) (Gabizon et al., 2008). Pegylated liposomal encapsulation reduces the plasma levels of free doxorubicin and may reduce drug delivery to normal tissues, which can in turn reduce cardiotoxicity (Gil-Gil et al., 2021). Results from clinical data revealed that PLD showed a similar efficacy to doxorubicin with a lower incidence of cardiotoxicity when administered in either advanced or (neo)adjuvant stages of treatment (O’Brien et al., 2004; Liu et al., 2021).

Human epidermal growth factor receptor 2 (HER2) is overexpressed in approximately 20–25% of breast cancer cases, which is associated with poor prognosis (Lynce et al., 2017). However, remarkable progress of trastuzumab plus chemotherapy as adjuvant treatment has significantly improved the survival rates of female breast cancer patients (Slamon et al., 2011; Goldhirsch et al., 2013; Perez et al., 2014; Cameron et al., 2017). Further development in the administration of dual anti-HER2 therapy with trastuzumab and pertuzumab shows great outcomes in (neo)adjuvant as well as metastatic settings (Baselga et al., 2012; Gianni et al., 2012; von Minckwitz et al., 2017). Trastuzumab is also associated with an increased risk of cardiotoxicity, particularly when administered in combination with anthracycline-based therapy. Cardiotoxicity, in this instance, would be manifested as symptomatic congestive heart failure (CHF) or asymptomatic decline of left ventricular ejection fraction (LVEF) (Seidman et al., 2002).

Previous studies have reported that 3–7% of patients who were receiving trastuzumab exhibited cardiac dysfunction in various forms (Seidman et al., 2002). When pertuzumab was combined with trastuzumab and chemotherapy, its cardiac safety was similar to that of trastuzumab alone (Swain et al., 2013; Yu et al., 2016). Nevertheless, limited data are available on the cardiac safety of PLD-based treatment administered sequentially with trastuzumab ± pertuzumab for the adjuvant treatment of HER2-positive early breast cancer (eBC) patients. Therefore, in this study, we aimed to explore strategies to reduce the cardiotoxicity of anthracycline sequential trastuzumab treatment and to clarify the cardiac safety and efficacy of PLD sequential trastuzumab treatment in adjuvant therapy.

## Patients and Methods

### Patients and Study Design

Baseline clinical data as well as posttreatment patient assessment data were retrospectively collected, including the LVEF, electrocardiogram (ECG) status, and efficacy, in patients with HER2-positive eBC treated with PLD-C (PLD plus cyclophosphamide) followed by TH (taxanes with trastuzumab) or TPH (taxanes with trastuzumab and pertuzumab) who then completed standard anti-HER2 treatment for a total of 12 months from June 2012 to August 2021.

### Eligibility

Inclusion criteria were patients with HER2-positive eBC diagnosed by pathology who had been treated with radical surgery, age of ≥18 years, and sequential use of PLD and trastuzumab ± pertuzumab. LVEF was evaluated at least twice at our center before and after treatment by 2D echocardiography.

Patients were excluded if they had metastatic disease or severe CHF (NYHA III–IV).

### Cardiac Monitoring

LVEF monitoring was performed before chemotherapy and every 3 months, where ECG including preanthracycline and pretrastuzumab was collected at multiple timepoints and sequentially throughout the therapy.

### Definition of Cardiotoxicity

Clinical cardiotoxicity caused by cancer therapy is defined as ① symptomatic CHF; ② an asymptomatic absolute decline of ≥16% from baseline LVEF; ③ an asymptomatic absolute decline of 10–15% from baseline LVEF to below the lower limit of normal (LLN = 50%); and ④ LVEF<45% (in these situations, antitumor therapy should be halted for more than 4 weeks, and LVEF needs to be rechecked at an interval of 3–4 weeks). Signs associated with CHF consist of S3 rhythm gallops and/or tachycardia (Yu et al., 2015). Subclinical cardiotoxicity is defined as ① an asymptomatic absolute decline of ≥10% and <16% from baseline LVEF and ② an asymptomatic absolute decline of <10% to below the LLN (LLN = 50%) (in these situations, antitumor therapy continued, and LVEF needs to be rechecked at an interval of 3–4 weeks) (Yu et al., 2015). Adverse events (AEs) were also monitored continuously and graded according to the National Cancer Institute Common Terminology Criteria for Adverse Events (NCI-CTCAE) v4.0.

### Interruption of Anti-HER2 Treatment and Efficacy Endpoint

Interruption of anti-HER2 treatment was defined as interruption of one or more doses or ≥6 weeks between doses.

The efficacy endpoint was disease-free survival (DFS), defined as the time from the first date of surgery to the first date of disease progression or August 2021.

### Adjuvant Treatment Plan and Dosage

Patients received PLD-C (PLD 25 mg/m^2^ on day 1 plus cyclophosphamide 600 mg/m^2^ on day 1) every 3 weeks for four cycles, followed by taxanes (paclitaxel 80 mg/m^2^ on day 1 or docetaxel 75–100 mg/m^2^ on day 1 or nab-paclitaxel 125 mg/m^2^ on day 1, 8) every 3 weeks for four cycles plus anti-HER2 therapy (trastuzumab was given at an initial dose of 8 mg/kg, followed by 6 mg/kg; pertuzumab was given at an initial dose of 840 mg, followed by 420 mg) every 3 weeks. Patients were allowed to complete 12 months of trastuzumab ± pertuzumab maintenance treatment until unacceptable toxicity levels were registered, or if/when there were signs of disease progression.

#### Statistical Analysis

The normally distributed continuous data were expressed as mean ± standard deviation. Qualitative data were expressed as frequency and percentage. Chi-square (χ^2^) test of significance was used to compare proportions between qualitative parameters. The Kaplan–Meier method was used to estimate the percentage of DFS at 2 and 5 years. A *p*-value of <0.05 was considered statistically significant. Data were analyzed using Statistical Program for Social Science version 25.0.

## Results

### Patient Characteristics

In total, 70 eligible patients were enrolled. The mean age was 54.0 years (range 30–70 years), and 63 patients (90.0%) were younger than 65 years. All patients underwent breast surgery, either modified radical mastectomy (88.6%) or conservative surgery (11.4%). Of note, 41 patients (58.6%) received radiation therapy, of which 20 patients (48.8%) received left chest wall radiotherapy. Among all the patients, 55 patients (78.6%) received PLD-C followed by TH and 15 patients (21.4%) received PLD-C followed by TPH. Of all cases, 14 patients (20.0%) had at least one cardiovascular risk factor, including hypertension, diabetes, or dyslipidemia. The concrete baseline characteristics of patients are listed in Table 1.

**TABLE 1:** Patients baseline characteristics (N = 70).

Patient characteristic	N (%)
Median age, years (range)	54 (30–70)
BMI (kg/m^2^)	23.0 ± 2.6
Histologic type	
Ductal carcinoma	65 (92.9)
Metaplastic carcinoma	3 (4.3)
Mucinous carcinoma	2 (2.9)
Tumor size (cm)	2.2 (0.5–11.0)
Histologically positive nodes, median (range)	1 (0–20)
0	29 (41.4)
1–3	27 (38.6)
≥4	14 (20.0)
Estrogen receptor status	
Positive	31 (44.3)
Negative	39 (5.7)
Progesterone receptor status	
Positive	22 (31.4)
Negative	48 (68.6)
Surgery type	
Mastectomy	62 (88.6)
Breast conserving	8 (11.4)
Radiotherapy	
Yes	41 (58.6)
No	29 (41.4)
Adjuvant regimens	
AC→T/DH	55 (78.6)
AC→ T/D/PHPt	15 (21.4)
Cardiovascular risk factors	
Yes	14 (20.0)
No	56 (80.0)

Numbers are mean ± standard deviation for continuous variables (unless otherwise specified), and N (%) for categorical variables; T: Paclitaxel; D: Docetaxel; P: nab-paclitaxel.

#### Cardiotoxicity Analysis

During the observation period, clinical cardiotoxicity occurred in six patients (8.6%), and all of them were observed to have an absolute decline of ≥16% from baseline LVEF but not below the LLN (LLN = 50%). Subclinical cardiotoxicity events occurred in 17 patients (24.3%), and all these patients had absolute declines of ≥10% and <16% from baseline LVEF but not below the LLN (LLN = 50%).

One patient developed syncope after the first cycle of PLD-C, and ECG studies further showed evidence of third-degree atrioventricular block, which was considered to be related to previous bradycardia. The patient underwent cardiac pacemaker implantation soon after but still completed the remaining seven cycles of chemotherapy and 1-year trastuzumab therapy. There was no cardiotoxicity manifested as a decrease in LVEF in this patient. No other patients were observed to have CHF, and there were no deaths caused by PLD or anti-HER2 drugs.

By August 2021, no patients were interrupted from treatment, and all patients completed anti-HER2 treatment for 12 months.

**FIGURE 1:**
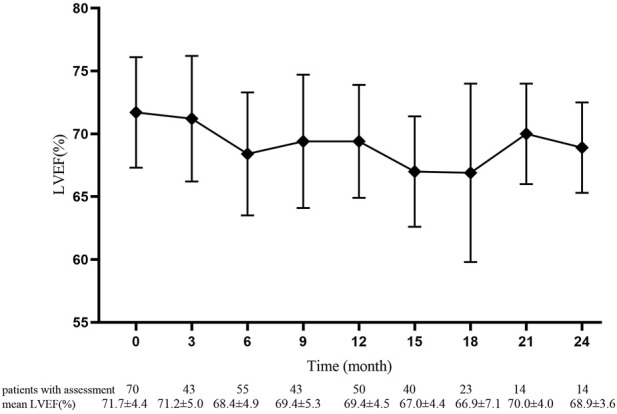
Pre-anthracycline baseline and value of left ventricular ejection fraction in 70 patients at each monitoring timepoint. Data are presented as mean ± standard deviation.

**FIGURE 2:**
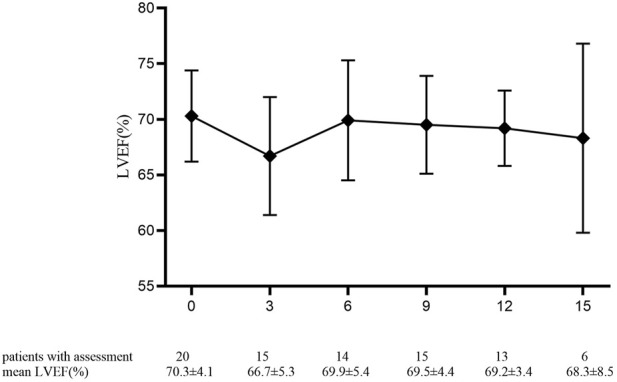
Pre-trastuzumab baseline and value of left ventricular ejection fraction percentage in 20 patients at each monitoring timepoint. Data are presented as mean ± standard deviation.

**FIGURE 3:**
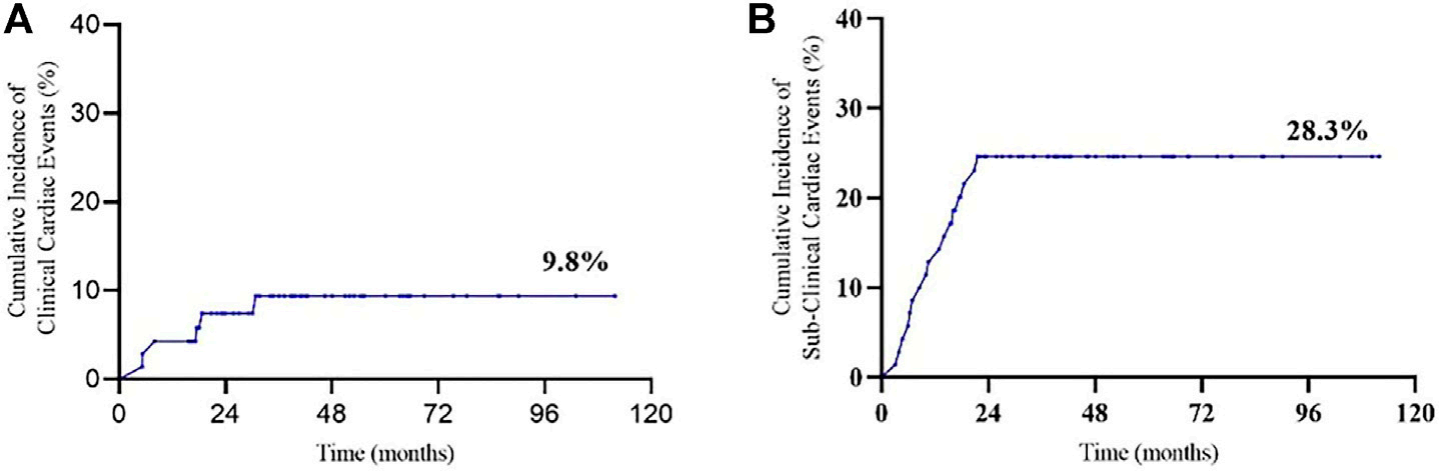
Cumulative incidence of cardiac events: **(A)** clinical cardiotoxicity and **(B)** subclinical cardiotoxicity.

The mean baselines of pre-AC and pre-H/HP LVEF were 71.7 ± 4.4% and 70.3 ± 4.1%, respectively. During the treatment period, the sharpest decrease in LVEF was observed within 18 months after the start of PLD treatment, which was equivalent to that within 15 months after the start of 12-month trastuzumab treatment (Figures 1 and 2). At each monitoring timepoint, the incidence of cardiotoxicity was the highest at the 18th month after the start of PLD or at the 15th month after the start of trastuzumab treatment, which was 8.7% (Table 2). LVEF began to recover at the 21st month after starting PLD treatment, which was the same as that observed at the 18th month after patients started trastuzumab treatment. The cumulative incidence of clinical cardiotoxicity-related events within 6 years was 9.8% and that of subclinical cardiotoxicity-related events within 6 years was 28.3% (Figure 3).

**TABLE 2:** Changes in left ventricular ejection fraction are divided into two groups, and the number of cases is listed.

△LVEF (LVEF-base)	3M(n = 43)	6M(n = 55)	9M(n = 43)	12M(n = 50)	15M(n = 40)	18M(n = 23)	21M(n = 14)	24M(n = 14)
10%∼15%	3 (7.0%)	2 (3.6%)	4 (9.3%)	0 (0)	4 (10.0%)	4 (17.4%)	0 (0)	0 (0)
≥16%	1 (2.3%)	2 (3.6%)	0 (0)	0 (0)	1 (2.5%)	2 (8.7%)	0 (0)	0 (0)

#### Survival Analysis

**FIGURE 4:**
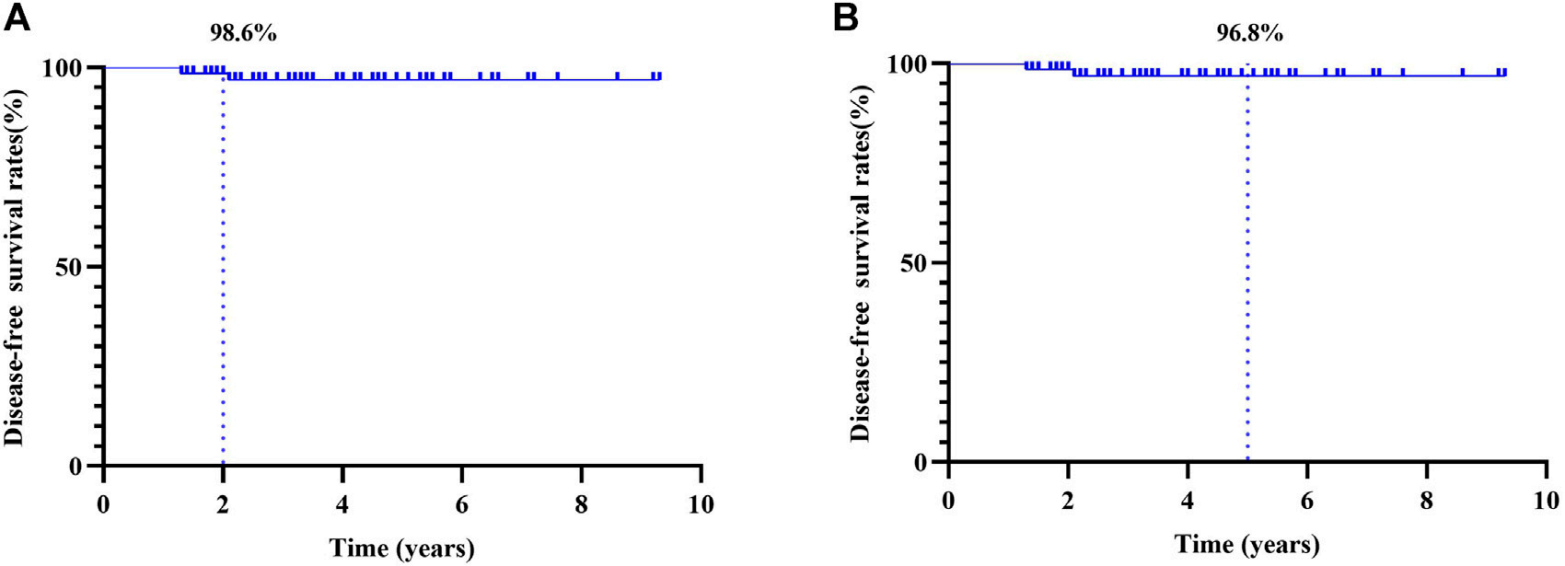
Kaplan–Meier curves for disease-free survival (DFS): **(A)** 2-year DFS and **(B)** 5-year DFS.

Until August 2021, only two patients developed local recurrent or metastatic diseases. In total, 57 patients were followed up for 2 years, and the 2-year DFS was 98.6%. Moreover, 18 patients were followed up for 5 years, and the 5-year DFS was 96.8% (Figure 4).

The main cardiac safety and efficacy results of this study were compared with other pivotal adjuvant treatments of eBC using anti-HER2 monoclonal therapy and summarized, as presented in Table 3.

**TABLE 3:** Summary of the cardiac safety and efficacy results of our study compared with other pivotal adjuvant trastuzumab trials.

Trial	Design	Median follow-up times	DFS	Severe CHF/CD	LVEF absolute decline from baseline	References
NCCTG N9831 (N = 1944)	arm B: AC (q3w x 4) →P (qw x 12)-H (qw x 52) arm C: AC (q3w x 4) →P (qw x 12) + H (qw x 12) →H (qw x40)	9.2-year	arm B: 80.1% (5-year) arm C: 84.5% (5-year)	arm B 2.8% arm C 3.7%	≥15%:arm B 14.6% ≥ 15%:arm C 18.7%	Advani et al. (2016)
NSABPB-31/N9831	AC→PH	8.4-year	73.7% (10-year)	2.0%	NR	Perez et al. (2014)
NSABP B-31 (N = 2043)	arm B: AC (q3w x 4) → P (q3w x 4 or qw x 12) + H (qw x 12) →H (qw x 40)	7-year	NR	arm B 3.9%	≥10%: 35.3% ≥ 15%: 14.6%	Romond et al. (2012)
BCIRG-006 (N = 3,222)	arm B: AC (q3w x 4) →TXT (q3w x 4) + H (qw x 12) → H (q3w x 13)	10-year	arm B: 84% (5-year) arm C: 81% (5-year)	arm B: 2.0% arm C: 0.4%	≥10%:18.6% ≥ 10%:9.4%	Slamon et al. (2011)
	arm C: Cb + TXT (q3w x 6) + H (qw x 18) →H (q3w x 12)					
HERA (N = 5,102)	arm B: A-based (A-T 26%; T-free 68%) → H (1-year q3w)	11-year	69% (10-year)	1%	≥10%:4.4%	Cameron et al. (2017)
Our study (N = 70)	PLD-C →TH/TPH (q3w) → finished H/PH treatment for a total of 12 months	3.5-year	98.6% (2-year) 96.8% (5-year)	0%	≥16%:8.6% ≥ 10% and<16:24.3%	

Abbreviations: A, doxorubicin; C, cyclophosphamide; P, paclitaxel; TXT, docetaxel; T, taxanes; H, trastuzumab; PH, pertuzumab and trastuzumab; CE, cardiac events; CD, cardiac death; carboplatin: Cb; CHF: congestive heart failure; DFS, disease-free survival; LVEF, left ventricular ejection fraction; NSABP, National Surgical Adjuvant Breast and Bowel Project; NCCTG, North Central Cancer Treatment Group; BCIRG, Breast Cancer International Research Group; HERA, herceptin adjuvant trial; qw, every week; q3w, every 3 weeks; NR, not reported. The chemotherapy regimens involved in these pivotal studies were all conventional doses.

#### Analysis of Factors Affecting Cardiotoxicity

In the univariate analysis, body mass index (BMI; <25, ≥25 kg/m^2^), age (<60, ≥60 years), left chest wall radiotherapy, and ongoing cardiovascular risk factors were not significantly associated with clinical or subclinical cardiotoxicity (*p* > 0.05); the results are listed in Tables 4 and 5.

**TABLE 4:** Univariate chi-square analyses for influencing factors of clinical cardiotoxicity according to patients’ characteristics.

Patient characteristics	Cardiotoxicity (n = 6)	No cardiotoxicity (n = 64)	χ2	P
Age			0.000	1.000
<60 years	4 (66.7%)	47 (73.4%)		
≥60 years	2 (33.3%)	17 (26.6%)		
Left chest wall radiotherapy			0.000	1.000
yes	2 (33.3%)	18 (28.1%)		
no	4 (66.7%)	46 (71.9%)		
Cardiovascular risk factors			0.103	0.749
yes	2 (33.3%)	12 (18.7%)		
no	4 (66.7%)	52 (81.3%)		
BMI			0.000	1.000
<25 kg/m^2^	5 (83.3%)	52 (81.3%)		
≥25 kg/m^2^	1 (16.70%)	12 (18.7%)		

**TABLE 5:** Univariate chi-square analyses for influencing factors of subclinical cardiotoxicity according to patients’ characteristics.

Patient characteristics	Cardiotoxicity (n = 17)	No cardiotoxicity (n = 53)	χ2	P
Age			0.000	1.000
<60 years	12 (70.6%)	35 (73.6%)		
≥60 years	5 (29.4%)	14 (26.4%)		
Left chest wall radiotherapy			0.157	0.692
yes	6 (24.3%)	14 (26.4%)		
no	11 (64.7%)	39 (73.6%)		
Cardiovascular risk factors			0.000	1.000
yes	3 (17.6%)	11 (20.8%)		
no	14 (82.4%)	42 (79.2%)		
BMI			0.000	1.000
<25 kg/m^2^	14 (82.4%)	43 (81.1%)		
≥25 kg/m^2^	3 (17.6%)	10 \(18.9%)		

#### Echocardiogram Parameter Changes

The most frequent cardiac disorder reported by echocardiogram was left ventricular diastolic dysfunction, which was more common in the no-cardiotoxicity group (70.3%) than in the cardiotoxicity group (66.7%). Left ventricular systolic dysfunction events had an incidence of 33.3% in the cardiotoxicity group (Table 6).

**TABLE 6:** Cardiac disorders (NCI-CTCAEv4.0, all grades).

Adverse event (n, %)	Cardiotoxicity (n = 6)	No cardiotoxicity (n = 64)
LVDD	4 (66.7%)	45 (70.3%)
LVSD	1 (16.7%)	1 (1.6%)
ST segment changes	0 (0.0%)	3 (4.7%)
Twave changes	5 (83.3%)	34 (53.1%)
ST-T segment changes	1 (16.7%)	14 (21.9%)
ST-T-U segment changes	2 (33.3%)	19 (29.7%)
T-U segment changes	1 (16.7%)	10 (15.6%)
Atrial premature beat ventricular premature beat	0 (0.0%) 0(0.0%)	9 (14.1%) 1 (1.6%)
Sinus tachycardia	0 (0.0%)	3 (4.7%)
Sinus bradycardia Sinus arrhythmia	3(50.0%) 0(0.0%)	5 (7.8%) 5 (7.8%)
Atrial fibrillation Cardiomegaly Bundle branch block right	0(0.0%) 0(0.0%) 0(0.0%)	0(0.0%) 0(0.0%) 8 (12.5%)
Extrasystoles	0 (0.0%)	0 (0.0%)
PR interval prolongation	0 (0.0%)	3 (4.7%)
QT interval prolongation	0 (0.0%)	2 (3.1%)

Abbreviations: LVSD, left ventricular systolic dysfunction; LVDD, left ventricular diastolic dysfunction.

#### Electrocardiogram Changes

In the cardiotoxicity group, ECG changes mainly included T-wave changes (83.3%), sinus bradycardia (50.0%), ST-TU segment changes (33.3%), ST-T segment changes (16.7%), and T-U segment changes (16.7%). The concrete disorders are listed in Table 6.

## Discussion

Over recent years, the application of anthracyclines sequentially with anti-HER2 therapy has shown to significantly improve the survival rates and prognoses of patients (Slamon et al., 2011). However, cardiotoxicity, as the major cause of breast cancer deaths, even without symptoms, may significantly limit the possibility of tumor treatment (Patnaik et al., 2011). In clinical practice, LVEF measured by echocardiography is used as an index to evaluate cardiotoxicity due to its accessibility.

PLD has been demonstrated to have equivalent efficacy but significantly less cardiotoxicity than conventional doxorubicin. In a phase III study, the cardiotoxicity rates were 3.9% and 18.8% with PLD and doxorubicin, respectively (O'Brien et al., 2004). A retrospective study compared the use of traditional anthracyclines and PLD in neoadjuvant therapy for patients with breast cancer. The study revealed higher pathologic complete response rates and lower incidences of cardiotoxicity in PLD-based cohorts (Liu et al., 2021). Therefore, it can be considered that the risk of cardiac toxicity after PLD sequential trastuzumab therapy is slightly higher than that of PLD monotherapy, but it is acceptable. Dual-target therapy with the addition of pertuzumab did not increase the incidence of cardiac-related AEs in either metastatic or neoadjuvant studies (Gianni et al., 2012; Schneeweiss et al., 2013).

PLD-C is not a standard protocol for breast cancer in adjuvant chemotherapy. However, due to the individualized treatment of cancer, some patients choose PLD as an alternative drug to anthracycline in adjuvant therapy. Through the narration of the physician in charge of these cases, the main reasons for these patients to choose PLD in our center include the following: 1) to avoid obvious hair loss caused by initial chemotherapy, 2) to avoid stronger nausea and vomiting symptoms, 3) to avoid the potential risk of developing febrile neutropenia, and 4) consideration of the potential to reduce the incidence of lifetime cardiotoxicity.

In this retrospective cohort study, PLD-C followed by anti-HER2 therapy as adjuvant therapy was effective and safe in a population of HER2-positive eBC patients. This study demonstrated that these schemes had a low risk for cardiotoxicity with slight clinical cardiotoxicity compared with treatments in previous clinical studies containing conventional anthracycline, taxanes, and trastuzumab (Table 3).

As described in Table 3, the NCCTG N9831 trial randomized patients between two arms in the adjuvant setting with AC followed by paclitaxel either with sequential or concurrent trastuzumab, and at a median follow-up of 9.2 years, the reported incidence of CHF or cardiac death (CD) was 2.8% in arm B (AC followed by paclitaxel and then trastuzumab) and 3.7% in arm C (AC followed by paclitaxel concurrent with trastuzumab) (Advani et al., 2016). In the NSABP-B31 study, at a median follow-up of 7 years, the incidence of severe CHF/CD was 3.9% in the arm with AC followed by TH. An independent retrospective review of the B-31 and N9831 trials demonstrated a 2.0% incidence of symptomatic CHF or CD in the trastuzumab-containing arm (Romond et al., 2012). In the BCIRG-006 study, at a median follow-up of 10 years, the incidence of CHF or CD was 2.0% in the arm with AC followed by docetaxel and trastuzumab (Slamon et al., 2011). While in our study, at a median follow-up of 3.5 years, the incidence of CHF or CD was 0% in the groups with PLD-C followed by TH/TPH (q3w). In the NCCTGN9831 and NSABPB-31 trials, LVEF reductions ≥15% from baseline were 14.6% (NCCTG N9831 arm B) and 18.7% (NCCTG N9831 arm C), respectively (Romond et al., 2012; Advani et al., 2016). While in our study, LVEF reduction ≥16% from baseline was 8.6%, where cardiotoxicity halved in value. Subclinical cardiotoxicity in the NSABP B-31 and BCIRG-006 studies using anthracyclines and trastuzumab was similar to that in our study (Slamon et al., 2011; Romond et al., 2012). This indicates that although PLD is used, cardiotoxicity monitoring is still necessary. In the HERA study, because 68% of patients did not use taxanes, the subclinical cardiotoxicity was relatively low (4.4%) (Cameron et al., 2017). In the NCCTGN9831 study, the 5-year DFS rates were 80.1% (arm B) and 84.5% (arm C) (Advani et al., 2016). In the BCIRG-006 study, the 5-year DFS rates were 84% (arm B:AC-TH group) and 81% (arm C:TCH group), respectively (Slamon et al., 2011). While in our study, the 5-year DFS was 96.8%, suggesting that patients had improved survival. It is worth noting that all patients completed PLD-C followed by TH or TPH every 3 weeks, which reflects the excellent tolerance; this may be related to higher DFS and efficiency.

The NCCTG N9831 trial demonstrated that age ≥60 years, registration LVEF <65%, and use of antihypertensive medications were associated with an increased risk of cardiac events, while radiation therapy, BMI, or ethnicity were not statistically significant (Advani et al., 2016). In the NSABP-B31 trial, LVEF (50–54%), age (≥60 years), and receiving antihypertensive medications and radiotherapy were also not statistically significant (Romond et al., 2012). The CANTO study indicated that obesity appeared to be associated with an important increase in risk-related cardiotoxicity in eBC patients (Kaboré and Guenancia, 2019). In our study, since the basal LVEF of the patients was not low and the incidence of cardiotoxicity was low, it was less possible to conclude that cardiotoxicity was related to BMI (<25, ≥25 kg/m^2^), age (<60, ≥60 years), radiotherapy, ongoing cardiovascular risk factors, or LVEF at baseline.

Our study had several limitations as follows. This study was a retrospective study, the test population was of a small sample size, and the LVEF values were not available for all patients at all corresponding observation points, leading to the possibility that the results showed a reduced incidence of cardiotoxicity. Due to the lack of necessary baseline or follow-up LVEF measurements, many patients had to be excluded, resulting in some selection bias. Therefore, as a future consideration, a prospective cohort study to further clarify the precise incidence and outcome of PLD sequential trastuzumab cardiotoxicity is worth carrying out.

## Conclusion

PLD sequential trastuzumab treatment reduces cardiotoxicity by nearly half compared with traditional anthracyclines, which is well tolerated. Most patients can complete the established treatment plans, which may have a positive effect on survival; this merits further exploration and may become an alternative strategy for cardioprotection in patients with HER2-positive eBC.

## Data Availability

The raw data supporting the conclusions of this article will be made available by the authors, without undue reservation.
